# Mosaic upd(14)pat in a patient with mild features of Kagami–Ogata syndrome

**DOI:** 10.1002/ccr3.1300

**Published:** 2017-11-28

**Authors:** Marte G. Haug, Atle Brendehaug, Gunnar Houge, Masayo Kagami, Tsutomu Ogata

**Affiliations:** ^1^ Department of Medical Genetics St Olav's Hospital Trondheim Norway; ^2^ Department of Medical Genetics Haukeland University Hospital Bergen Norway; ^3^ Department of Molecular Endocrinology National Research Institute for Child Health and Development Tokyo Japan; ^4^ Department of Pediatrics Hamamatsu University School of Medicine Hamamatsu Japan

**Keywords:** Kagami–Ogata syndrome, mild phenotype, mosaicism, upd(14)pat

## Abstract

We report a Norwegian girl with mild clinical features of Kagami–Ogata syndrome (KOS) and mosaic upd(14)pat. To our knowledge, this is the first report describing a mosaic patient with KOS. These results imply that mosaic uniparental disomy should be examined in patients with mild features of imprinted disorders.

## Introduction

Kagami–Ogata syndrome (KOS) (OMIM #608149) is a rare imprinting disorder primarily caused by paternal uniparental disomy 14 (upd(14)pat) and epimutations (hypermethylations) and microdeletions affecting the germline‐derived *MEG3*/*DLK1*:IG‐DMR (differentially methylated region) and the postfertilization‐derived *MEG3*:TSS‐DMR of maternal origin 1. KOS is clinically characterized by unique facial appearance, small bell‐shaped thorax with coat‐hanger appearance of the ribs, abdominal wall defects, placentomegaly, and polyhydramnios 1. In particular, unique facial appearance with full cheeks and protruding philtrum and distinctive chest roentgenograms with increased coat‐hanger angles (CHA) to the ribs constitute the pathognomonic features from infancy through childhood, while the ratio of mid to widest thorax diameter (M/W ratio), although it is definitely below the normal range in infancy, becomes within the normal range after infancy. In addition, body growth is fairly preserved, and developmental delay and/or intellectual disability is invariably observed 1. As the constellation of such clinical findings is highly specific to KOS, this permits clinical diagnosis of KOS. However, clinical findings could be mild in mosaic patients with normal cells. Here, we report mosaic upd(14)pat identified in a patient with mild KOS phenotype.

## Case Presentation

This Norwegian girl was conceived naturally and was delivered by Caesarean section at 38 weeks of gestation due to fetal distress. Her prenatal course was complicated by mild polyhydramnios from ~28 weeks of gestation, although amnioreduction was not required. Placental size was unknown. The 35‐year‐old father and the 33‐year‐old mother were nonconsanguineous and clinically normal.

At birth, her length was 48.0 cm (−1.2 SD), and her weight 3.07 kg (−1.2 SD) 2. Apgar score was 6 at 1 min and 7 at 5 min. She was admitted to neonatal intensive care unit due to respiratory distress and was placed on nasal continuous positive airway pressure therapy for 24 h; mechanical ventilation was not required. She also received tube feeding for 8 days because of poor sucking. Physical examination in infancy revealed a somewhat characteristic face and short webbed neck (Fig. S1A). She had umbilical hernia that was surgically treated at 3 years of age. Subsequently, while she was diagnosed as having Asperger syndrome at 3.5 years of age, she was apparently free from developmental delay and was enrolled in a regular class. On the latest physical examination at 13 years of age, her height was 166.5 cm (+1.1 SD), her weight 58.0 kg (+1.6 SD), and her head circumference 55.5 cm (+1.1 SD) 2. She still exhibited somewhat characteristic facial appearance and had large hands and feet and joint contractures (Fig. S1B).

As clinical findings of this patient were suggestive of the presence of an underlying genetic cause, we performed genetic studies. This study was performed according to the Norwegian regulations and under approval of the Japanese Institutional Review Board Committees. We obtained written consent for the genetic studies and the publication of the photographs of the patient.

**Figure 1:**
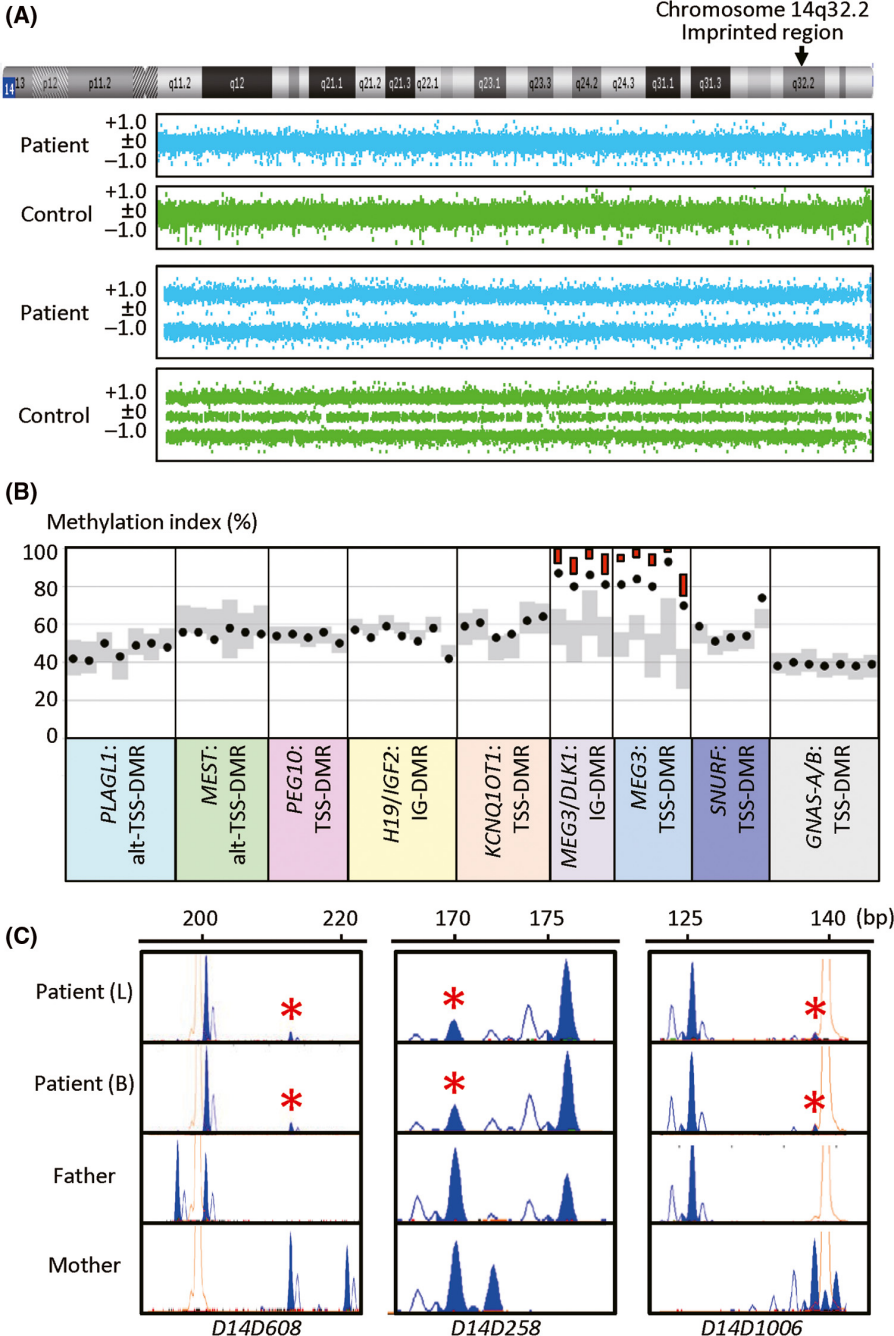
Representative molecular findings. (A) Array CGH and SNP array analyses for chromosome 14. The upper two panels show array CGH findings. The vertical axis shows log_2_ signal ratios, and the signals around +0.5, ±0, and −1.0 indicate duplicated, normal, and deleted segments, respectively. A region with ≥three consecutive dots that are present in increased or decreased log_2_ signal ratios is regarded as a copy number variant, and there is no such a region in this patient as well as in a control subject. The lower two panels show the SNP array findings. Most SNPs are present in a homozygous status (+1.0 or −1.0), and a small number of SNPs are apparently present in a heterozygous status (±0) in this patient, whereas examined SNPs are present in both homozygous and heterozygous conditions in the control subject. (B) Methylation indices (MIs, the frequencies of methylated clones) of the CpG sites within nine DMRs examined by pyrosequencing. The black circles show the MIs of this patient. Gray vertical bars indicate the ranges of MIs (minimum–maximum) in 50 control subjects, and the red bars for the *MEG3*/*DLK1*:IG‐DMR and *MEG3*:TSS‐DMR represent the range of MIs (minimum–maximum) in 11 KOS patients with upd(14)pat. (C) Microsatellite analysis. Major peaks of paternal origin and minor peaks of maternal origin (red asterisks) are identified in this patient. For *D14S258*, while the minor peak could be of paternal or maternal origin, overall SNP array data and microsatellite data indicate that the minor peak is of maternal origin (see Note S1). L: leukocytes, and B: buccal cells.

We first performed conventional cytogenetic analysis, in parallel with comparative genomic hybridization (aCGH) and SNP array analyses for leukocyte genomic DNA (gDNA) using Cytoscan HD (Affymetrix, Santa Clara, CA). Cytogenetic studies showed a normal 46,XX karyotype in two lymphocyte metaphase spreads examined, and aCGH and SNP array analyses revealed the absence of pathogenic copy number variant and the presence of apparent but not complete isodisomy for whole chromosome 14 (iso‐upd(14)pat) with a small number of heterozygous SNPs (Fig. 1A). Thus, we next carried out pyrosequencing analysis for *MEG3*/*DLK1*:IG‐DMR and *MEG3*:TSS‐DMR as well as other seven DMRs related to imprinting disorders with PyroMark Q24 (Qiagen), using leukocyte gDNA of the patient (for primers, see Table S1). Consequently, we showed isolated hypermethylations of the *MEG3*/*DLK1*:IG‐DMR and *MEG3*:TSS‐DMR, with the methylation indices for the two DMRs being higher than those of 50 control subjects and lower than those of 11 KOS patients with upd(14)pat (Fig. 1B). We further performed microsatellite analysis for 10 loci on chromosome 14, using leukocyte gDNA of the patient and the parents (for primers, see Table S2), demonstrating the presence of cells with maternally inherited chromosome 14 (Fig. 1C and Table S2). Collectively, these findings indicated the presence of mosaicism with iso‐upd(14)pat cells and normal cells in this patient, and the ratio of iso‐upd(14)pat cells to normal cells was calculated as ~73%:27% in the leukocyte gDNA and ~66%:34% in the buccal cell gDNA of the patient, using the data for *D14S258* (Note S1).

**Figure 2:**
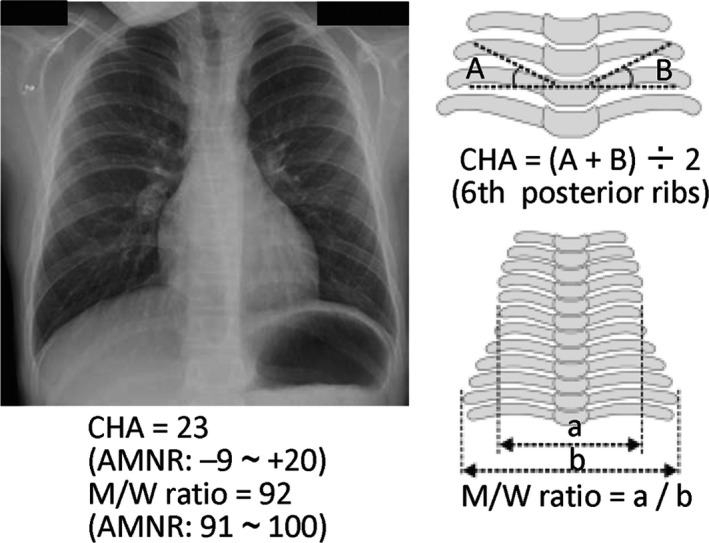
Chest roentgenogram at 9 years of age. AMNR: age‐matched normal range in the Japanese children 3.

After the identification of mosaic upd(14)pat, clinical features of this patient were re‐evaluated on the basis of the recently defined characteristic phenotypes in KOS 3. Consequently, it was found that although KOS‐like facial features such as depressed nasal root, anteverted nares, full cheeks, protruding philtrum, and puckered lips were identified in infancy, such facial “gestalt” was less prominent in this patient (Fig. S1A). Similarly, although such KOS‐like facial features were also exhibited at 13 years of age, full cheeks were barely recognized (Fig. S1B). Furthermore, KOS‐like thoracic and/or rib abnormalities were not recorded for chest roentgenograms obtained in the early neonatal period (unfortunately, roentgenograms were not preserved), and chest roentgenograms obtained at 9 years of age for a minor chest trauma showed that the CHA was slightly above the normal range and the M/W ratio remained within the normal range (Fig. 2). Thus, together with the lack of severe polyhydramnios, neonatal respiratory dysfunction, and developmental delay, clinical features of this patient were assessed as obviously milder than those of the previously reported patients with KOS 3, as predicted from the mosaicism with normal cells.

## Discussion

**Figure 3:**
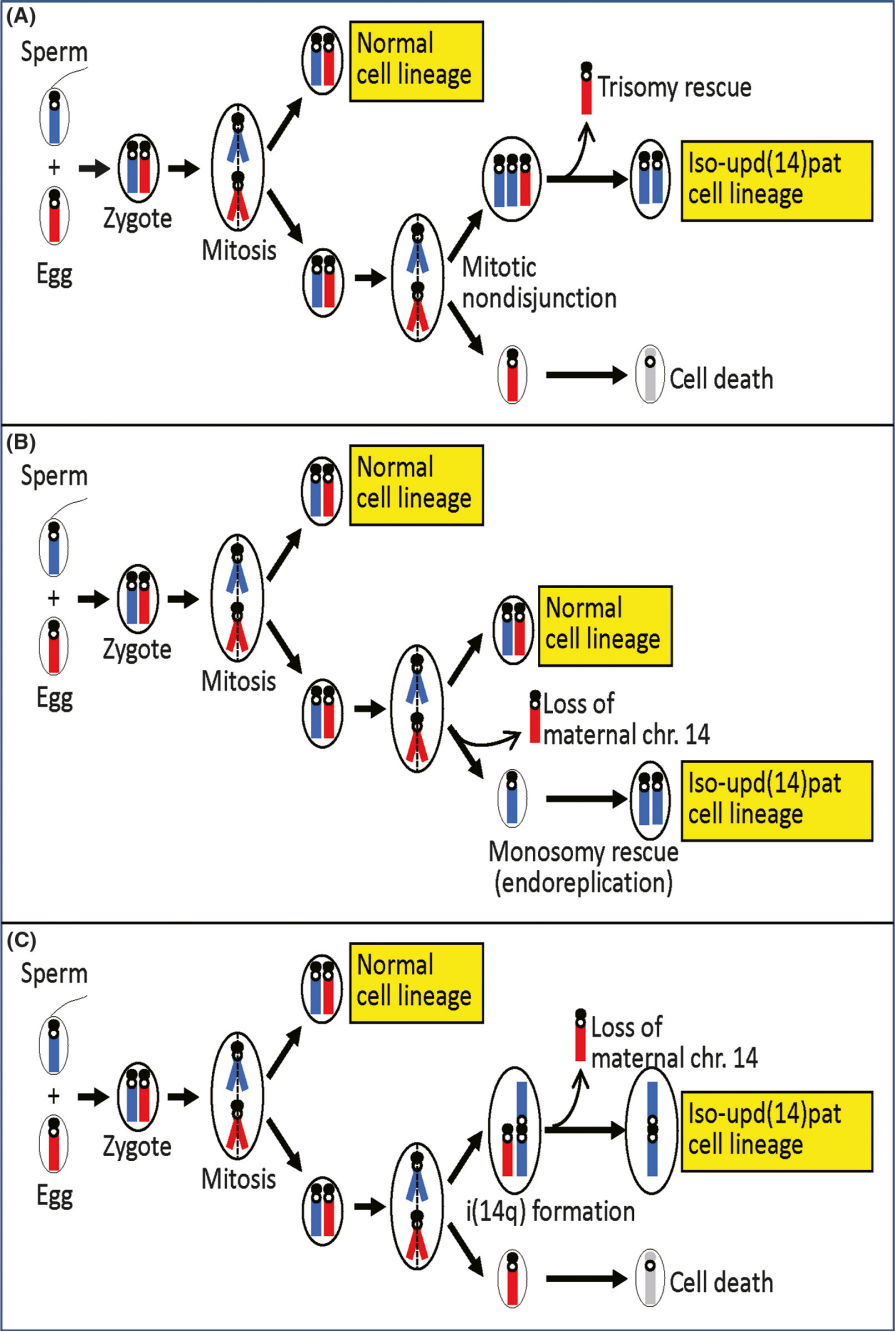
Schematic representation of the generation of the mosaic iso‐upd(14)pat. The paternally and maternally derived chromosome 14 homologs are shown in blue and red, respectively.

We identified mosaic iso‐upd(14)pat (full isodisomy) in a patient with mild KOS‐like features. Such mosaicism with a normal karyotype (46,XX/46,XX,upd(14)pat) would be generated by either mitotic nondisjunction and subsequent trisomy rescue (loss of a maternally transmitted chromosome 14 from a trisomic cell; Fig. 3A) or loss of a maternal chromosome 14 during mitosis followed by monosomy rescue (endoreplication of a paternally inherited chromosome 14 in a monosomic cell; Fig. 3B). In this regard, although monosomic cells would be lethal and eliminated from the body, trisomic cells might be present in the patient. Indeed, trisomic cells with two paternally or maternally inherited chromosome 14 homologs have previously been detected in patients with severe clinical features 4, 5. However, the mild phenotype of this patient and the aCGH finding suggest that such trisomic cells would remain quite minor, if any. Furthermore, it might also be possible that i(14q) chromosome formation took place in association with loss of chromosome 14 of maternal origin, resulting in the development of 45,XX, i(14q) with paternal uniparental disomy (Fig. 3C). In this regard, it is assumed that the i(14q) chromosome remained undetected, because karyotype was examined only in two lymphocytes.

Such mosaicism consisting of cells with full uniparental isodisomy and those with normal karyotype might not be so rare. Indeed, previous studies have identified 46,XY/46,XY,upd(7)mat (the frequency of iso‐upd(7)mat cells: 92% in leukocytes and 91% in salivary cells), 46,XX/46,XX,upd(11)mat (the frequency of iso‐upd(11)mat cells: 18% in leukocytes), and 46,XY/46,XY,upd(11)mat (the frequency of iso‐upd(11)mat cells: 20% in leukocytes and 74% in buccal cells) in three patients with Silver–Russell syndrome‐like phenotype 6, 7, 8. Furthermore, such mosaicism might have remained undetected in a certain fraction of patients, because (1) upd cells could not be detected when they are barely present in leukocytes that are usually utilized for genetic analyses and (2) genetic studies may not be performed in mosaic patients with mild clinical features. Further studies will provide useful information on the frequency and phenotypic spectrum of patients with mosaic uniparental disomy.

## Conclusion

We observed mosaic upd(14)pat in a patient with mild KOS phenotype. Thus, we recommend performing molecular studies to detect mosaicism in patients with mild imprinting disorder‐like phenotypes.

## Conflict of Interest

The authors declare no conflict of interest.

## Authorship

MGH: involved in the patient care, developing the idea, and drafting the article. AB, GH, and KM: performed molecular analyses. TO: coordinated the study, supervised the molecular studies, and wrote the paper.

## Supporting information


**Table S1.** Primers utilized in the pyrosequencing analysis.
**Table S2.** The primer sequences and the results of microsatellite analysis.**Figure S1.** Facial photographs at 1 year of age (A) and at 13 years of age (B).
**Note S1.** Calculation of the ratio of iso‐UPD(14)pat cells in leukocytes and buccal cells, using the microsatellite data for *D14S258*
