# Supplementary material for: Mosaic upd(14)pat in a patient with mild features of Kagami–Ogata syndrome
Source: Clin Case Rep. 2017 Nov 28;6(1):91–5. doi: 10.1002/ccr3.1300 (PMC5771875; doi:10.1002/ccr3.1300)
Supplement: Supplementary file 1 — Table S1. Primers utilized in the pyrosequencing analysis. Table S2. The primer sequences and the results of microsatellite analysis.Figure S1. Facial photographs at 1 year of age (A) and at 13 years of age (B). Note S1. Calculation of the ratio of iso‐UPD(14)pat cells in leukocytes and buccal cells, using the microsatellite data for D14S258 [file CCR3-6-91-s001.pdf]

Supplementary Materials

Mosaic UPD(14)pat in a Patient with Mild Features of Kagami-Ogata Syndrome

Marte G. Haug,[1] Atle Brendehaug,[2] Gunnar Houge,[2]
Masayo Kagami,[3] Tsutomu Ogata[3,4]

[1] Department of Medical Genetics, St Olav's Hospital, Trondheim, Norway

[2] Department of Medical Genetics, Haukeland University Hospital, Bergen, Norway

[3] Department of Molecular Endocrinology, National Research Institute for Child Health and Development, Tokyo, Japan

[4] Department of Pediatrics, Hamamatsu University School of Medicine, Hamamatsu, Japan.

Table S1. Primers utilized in the pyrosequencing analysis.

	Forward (5’ $\rightarrow$ 3’)	Reverse (5’ $\rightarrow$ 3’)	Sequence primer	AT	PS	CN
PLAGL1 :alt-TSS-DMR	GGGGTAGTYGTGTTTATAGTTTAG	biotin–CCCAAACACCTACCCTAC	GGGTAGTYGTGTTTATAGTTTAGT	55	146	45
6q24.2	ch6:144329336–144329359	ch6:144329214–144329231	ch6:144329335–144329358			
PEG10 :TSS-DMR	AGAAATTTGATTGYGTTTTGAGGAGAAT	ACCTTTAAAACTTAATTTCCCCATTTAT	AGTTTGGYGAAAGGTT	55	344	45
7q21.3	chr7:94285716–94285743	chr7:94286028–94286059	chr7:94285762–94285777			
MEST :alt-TSS-DMR	GTGTGGTTGGYGGTTTTGGGATTA	biotin–ACACCCCCTCCTCAAATA	TGTTTTTGGGYGAAAATTTTAT	55	143	45
7q32.2	ch7:130132206–130132229	ch7:130132332–130132348	ch7:130132276–130132297			
H19 /*IGF2* :IG-DMR	TTTGGGAGAGTTTGTGAGG	biotin–CCCCAAACCRATTCCCATCCAATTA	GRTAATATGYGGTTTTTAGATAGG	56	203	45
11p15.5	chr11:2019763–2019781	chr11:2019689–2019712	chr11:2019579–2019603			
KCNQ1OT1 :TSS-DMR	GGATTTAGAATTAYGATGYGGATTTTA	biotin–TCCCATCTACACCTTATAAACA	TTTTGAATTATTATGAGAATTATAG	55	155	45
11p15.5	ch11:2720333–2720359	ch11:2720466–2720487	ch11:2720383–2720407			
MEG3 /*DLK1* :IG-DMR	ATTTGGTATTTGTAGTTTTATGTTAAGATG	biotin–AATCAAAACAACTCAAATCCTTTATAAC	AATTGGGTTTGTTAGTAG	54	164	45
14q32.2	ch14:101275613–101275642	ch14:101275749–101275776	ch14:101275685–101275702			
MEG3 :TSS-DMR	TTGTGTTTGAATTTATTTTGTTT	biotin–CCCCAAATTCTATAACAAATTACTCT	GTGTTTGAATTTATTTTGTTT	54	167	45
14q32.2	ch14:101292170–101292192	ch14:101292311–101292336	ch14:101292172–101292192			
SNURF :TSS-DMR	GGGATATTTGAGATTTTGAAAGAA	biotin–AATACAAAACCTCCCCTACT	GTTATTTTTTTTATTTGGGAGGA	54	110	45
15q11.2	ch15:25200788–25200813	ch15:25200823–25200837	ch15:25200880–25200897			
GNAS-A/B :TSS-DMR	GGGATATTTGAGATTTTGAAAGAA	biotin–AATACAAAACCTCCCCTACT	GTTATTTTTTTTATTTGGGAGGA	52	217	45
20q13.32	chr20:57463531–57463555	chr20:57463727–57463746	chr20:57463630–57463652			

AT: annealing temperature (°C); PS: product size (bp); CN: cycle numbers; Y: C or T (pyrimidine); and R: A or G (purine).

Physical positions of the primers are based on the NCBI database (Genome Build 37.1).

Table S2. The primer sequences and the results of microsatellite analysis.

Locus	Position	Forward primer (5' → 3')	Reverse primer (5' → 3')	Mother	Patient	Father	Assessment
D14S608	14q12	TAAAGGTTTATCCATGCTGTAGC	ACGTGGTACAGGTAGATAAATGG	214/222	202/214*	198/202	iso-UPD(14)pat (mosaic)
D14S63	14q23.2	TTTACTCTCACTGCCCTG	AATGGTCCAGTCTGCC	206	206*/210	210/216	iso-UPD(14)pat (mosaic)
D14S588	14q24.2	GCCGAAAGAAAGAAAAAAGG	CGAATGCATACTTGCTGTTG	114/118	114*/118	114/118	Not informative
D14S258	14q24.2	TCACTGCATCTGGAAGCAC	CTAACTAAATGGCGAGCATTGAG	170/172	170*/176	170/176	Not informative
D14S267	14q32.12	TTAATGCCCACTGAATGCT	AAGGCAGCCCTGGTTT	213/219	207/213*	207/215	iso-UPD(14)pat (mosaic)
D14S617	14q32.12	TTTTAGGTGGCCACCATCTA	CCAGTTTAGGCAACAGAACA	141/163	141/163*	141/167	Not informative
D14S1006	14q32.2	TTCCACAGGGCAAGCAGTA	TTCTGGCAAAACCCAACC	138/140	126/138*	126	iso-UPD(14)pat (mosaic)
D14S985	14q32.2	CAGTGTGACCTTAAACAAGTCG	CCTGTGGGGTAGATACACGA	128/138	128*/134	134	iso-UPD(14)pat (mosaic)
D14S292	14q32.33	CTGTGTGGTGCATCAATG	CATGAAGGCAGCCTCA	107/109	109	109	Not informative
D14S1007	14q32.33	AGCTCCTATATGTCTTCACACAG	CTCCATTCCCATACGTCC	110/122	122*/124	124	iso-UPD(14)pat (mosaic)

The Arabic numbers represent the PCR product sizes in bp.

The *MEG3* /*DLK1* :IG-DMR and *MEG3* :TSS-DMR reside between *D14S985* and *D14S292* .

* Minor peaks.

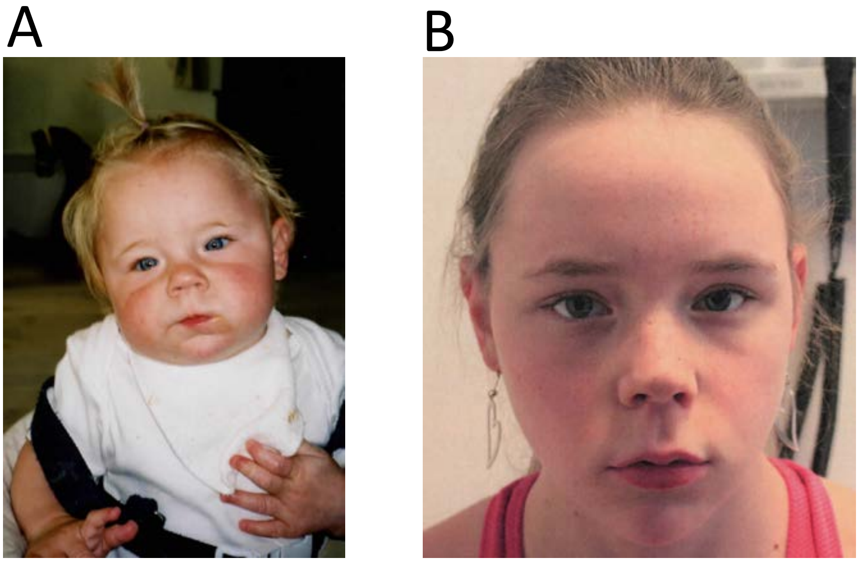

Fig. S1. Facial photographs at one year of age (A) and at 13 years of age (B).

SUPPLEMENTARY NOTE

Calculation of the ratio of iso-UPD(14)pat cells in leukocytes and buccal cells, using the microsatellite data for *D14S258*

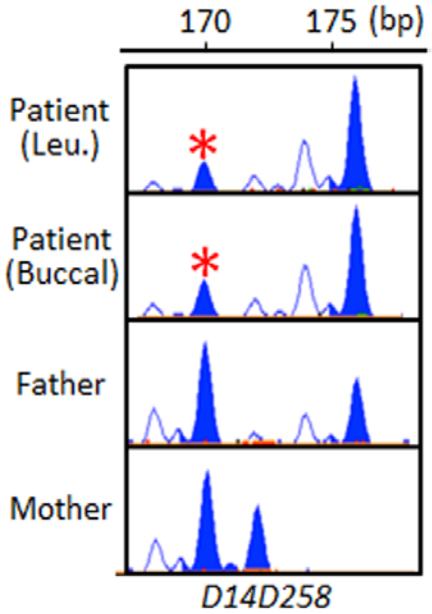

Molecular studies have indicated the presence of cells with isodisomy for whole chromosome 14 and those with biparentally inherited chromosome 14 homologs in this patient. Thus, the genotyping data for *D14S258* argue that the major 176 bp peak is derived from the father and the minor 170 bp is transmitted from the mother (highlighted with red asterisks). In the father who is also heterozygous for the identical two peaks, the area under curve (AUC) is larger for the short 170 bp peak than for the long 176 bp peak. This unequal amplification is consistent with short products being more easily amplified than long products. In the patient, the AUC ratio between the two peaks is obtained as 1.0:6.5 for leukocytes and 1.0:4.9 for buccal cells, after compensation of the unequal amplification between the two peaks using the paternal data.

Here, let "X" represent the frequency of the 46,XX,upd(14)pat (isodisomy) cells in leukocytes (thus, (1 – X) denotes the frequency of normal 46,XX cells in leukocytes). Then, the maternally derived 170 bp peak is generated by one maternally derived chromosome in the normal 46,XX cells, i.e., (1 – X), and the paternally derived 176 bp peak is formed by the products from two paternally derived homologous chromosomes in the 46,XX,upd(14)pat cells and one paternally derived chromosome in the normal 46,XX cells, i.e., (2X + (1 – X)) = (X + 1). Thus, the AUC ratio between the two peaks is represented as (1 – X):(X + 1) = 1.0:6.5, and "X" is obtained as 0.73 (73%). Similarly, when "Y" represents the frequency of the 46,XX,upd(14)pat cells in salivary cells, "Y" is obtained as 0.66 (66%).
